# Perceived Interpersonal Discrimination and Older Women’s Mental Health: Accumulation Across Domains, Attributions, and Time

**DOI:** 10.1093/aje/kwx326

**Published:** 2017-10-04

**Authors:** Laia Bécares, Nan Zhang

**Affiliations:** 1 Cathie Marsh Institute for Social Research, University of Manchester, Manchester, United Kingdom; 2 Department of Social Statistics, School of Social Sciences, University of Manchester, Manchester, United Kingdom

**Keywords:** cumulative disadvantage, discrimination, mental health, race/ethnicity, women

## Abstract

Experiencing discrimination is associated with poor mental health, but how cumulative experiences of perceived interpersonal discrimination across attributes, domains, and time are associated with mental disorders is still unknown. Using data from the Study of Women’s Health Across the Nation (1996–2008), we applied latent class analysis and generalized linear models to estimate the association between cumulative exposure to perceived interpersonal discrimination and older women’s mental health. We found 4 classes of perceived interpersonal discrimination, ranging from cumulative exposure to discrimination over attributes, domains, and time to none or minimal reports of discrimination. Women who experienced cumulative perceived interpersonal discrimination over time and across attributes and domains had the highest risk of depression (Center for Epidemiologic Studies Depression Scale score ≥16) compared with women in all other classes. This was true for all women regardless of race/ethnicity, although the type and severity of perceived discrimination differed across racial/ethnic groups. Cumulative exposure to perceived interpersonal discrimination across attributes, domains, and time has an incremental negative long-term association with mental health. Studies that examine exposure to perceived discrimination due to a single attribute in 1 domain or at 1 point in time underestimate the magnitude and complexity of discrimination and its association with health.

Discrimination—the treatment of someone less favorably than another person because of an identity group characteristic—has been shown to have a negative association with mental health (1–3). The majority of studies assessing whether discrimination leads to mental disorders have focused on examining how general mistreatment or race-based discrimination is adversely associated with mental health (4), although some other attributes, such as weight (5, 6), age (7, 8), sex (9), and sexual orientation (10, 11), have also received attention. Regardless of the attribution of perceived discrimination, most studies to date have examined how isolated attributes are associated with mental health. Identifying single sets of health determinants, such as unique forms and attributions of perceived discrimination, strips away the context of people’s lives and disregards the fact that individuals often embody more than 1 socially disadvantaged status and that these statuses and multiple experiences of discrimination interact to shape people’s health and life chances (12).

Theories of intersectionality argue that multiple marginalizations are interlinked and operate simultaneously (13, 14) and can therefore not be understood by theoretical and empirical approaches that treat each marginalized identity as an independent subject of inquiry. Instead, examinations of the intersections between social identities (including race/ethnicity, sex, class, sexuality, indigeneity, and disability/ability, among others) and forms of systemic oppression (including racism, classism, sexism, ableism, and homophobia) provide a more nuanced understanding of the social processes that generate and reproduce poor mental health and health inequities.

Excluding some exceptions (10, 15–27), the association between experiencing multiple forms of perceived discrimination due to intersecting social identities and mental health has rarely been explored. The few studies that have examined multiple types of discrimination have shown, for the most part, that experiencing numerous forms of discrimination has a greater negative association with mental health than experiencing discrimination due to 1 attribute only (10, 15–18, 20, 21). Although these studies bridge an important gap in the knowledge base about the mental health implications of experiencing discrimination due to multiple attributes, there are several important limitations to this work. For example, to our knowledge, none of these studies have examined how, in addition to the accumulation of attributions of discrimination, the accumulated experiences of discrimination over time and across multiple domains combine to affect mental health. This is important because examining single attributes, during a cross-section of people’s lifetimes or averaging across years, and exploring discrimination in 1 domain only fails to adequately capture marginalized people’s lived experiences and underestimates the harmful effect of discrimination on health.

Studies of racial discrimination show a clear dose-response relationship between increasing number of domains of discrimination experienced and incremental worsening of health (28–31). The information these studies provide is vital to our understanding of the extensiveness of discrimination in people’s lives, although these dose-response studies have not taken into account how this pervasiveness is associated with health over time. In a recent study, Wallace et al. (32) explored the role of cumulative discrimination in health across time and domains, reporting that the accumulation of experienced racial discrimination over time and across several domains has an incremental negative long-term association with psychological distress. That study provided novel information on the importance of considering cumulative experiences of discrimination over time and domains, but it examined only racial discrimination and disregarded the accumulation of discrimination due to multiple attributes.

We aimed to combine these 2 bodies of literature (the accumulation of experienced discrimination across attributes and across time and domains) to examine the cumulative association with mental health of the multiple forms of oppression that individuals experience over time. We did this by examining the associations that intersecting experiences of perceived interpersonal discrimination across domains, attributes, and time had with mental health in a multiethnic cohort of older women in the United States.

## METHODS

### Data and measures

For this analysis, we used publicly available data from the Study of Women’s Health Across the Nation (SWAN), a community-based, multisite, longitudinal study of the menopausal transition. The study design and recruitment for SWAN have been described elsewhere (33). Briefly, SWAN recruited 3,302 women drawn from 7 cities across the United States. All sites enrolled non-Hispanic white participants, and each site also enrolled women of either African-American, Japanese-American, Chinese-American, or Hispanic racial/ethnic background. At study entry, participants were between the ages of 42 and 52 years, self-identified as a member of one of the designated racial/ethnic groups or as white, reported recent menses (<3 months prior to enrollment), were either premenopausal or early perimenopausal, and were not using hormone replacement therapy. Study questionnaires were translated into Cantonese, Japanese, and Spanish. Institutional review board approval was granted, and informed consent was obtained from each study participant.

In this study, we used complete data from women who participated in SWAN from the baseline interview through wave 10 (1996–2008; *n* = 1,613) and belonged to the black or African-American (*n* = 411), Chinese-American (*n* = 183), Japanese-American (*n* = 182), or non-Hispanic white (*n* = 837) racial/ethnic group. Hispanic participants were excluded because of small sample sizes.

### Mental health

Mental health was measured using the 20-item Center for Epidemiologic Studies Depression Scale (34), a measure of depressive symptomatology. Respondents were asked how often in the past week they had experienced several symptoms, including feeling depressed or feeling like everything was an effort. Response categories ranged from 0 (rarely/none of the time) to 3 (most/all of the time). Positive statements were reversed so that higher scores reflected more depressive symptoms. Center for Epidemiologic Studies Depression Scale score was modeled as a dichotomous variable following the threshold of 16 (score ≥16 = depressed), and depression was measured at baseline and wave 10.

### Experiences of perceived interpersonal discrimination

Participants completed an adapted 10-item version of the Everyday Discrimination Scale (35), which uses a 4-point scale (1 = often, 4 = never) to assess the frequency of experiences of perceived interpersonal discrimination in respondents’ day-to-day lives. Each item starts with the following question: “In your day-to-day life, have you had the following experiences?” Example response items include “You are treated with less courtesy than other people” and “You receive poorer service than other people at restaurants or stores.” The original 9-item measure was modified in the SWAN study protocol to include one additional item, “People ignore you or act as if you are not there.”

Each of the 10 domains of perceived interpersonal discrimination was dichotomized into 0 (never) or 1 (rarely, sometimes, or often). The Everyday Discrimination Scale provides options for indicating attributions of perceived interpersonal discrimination, which include race, age, sex, physical appearance, ethnicity, income level, sexual orientation, language, and other. Due to problems with small cell sizes, we combined responses regarding the attributes of race and ethnicity into “race/ethnicity” and responses regarding age, physical appearance, income level, sexual orientation, language, and other attributes into “other.” Sex was left as a single attribute. Responses to the Everyday Discrimination Scale were reverse-scored so that higher numbers indicated greater unfair treatment. Perceived interpersonal discrimination across domains and attributes was assessed over 6 waves of SWAN (baseline and waves 1, 2, 3, 7, and 10).

### Covariates

Factors thought to be associated with both experiences of perceived interpersonal discrimination and mental health were considered in analytical models. These included age (years; continuous), marital status (single, never married; married; or separated, divorced, or widowed), nativity (born in the United States or born abroad), occupation (professional, nonmanual worker, skilled manual worker, or semiskilled/unskilled manual worker), and education (less than high school, high school graduate, some college, college graduate, or postgraduate education). Covariates were assessed at baseline.

### Analysis plan

The accumulation of perceived interpersonal discrimination across attributes, domains, and time was captured with longitudinal latent class analysis, a person-centered approach that probabilistically assigns individuals to latent classes based upon similar patterns of observed longitudinal data. Latent class analysis was first used to evaluate the fit of a 2-class model, and we systematically increased the number of classes in subsequent models until the addition of latent classes did not further improve model fit. For each model, replication of the best log-likelihood was verified to avoid local maxima. To determine the optimal number of classes, we compared models across several model fit criteria. First, we evaluated the sample-size-adjusted Bayesian Information Criterion (36); lower relative Bayesian Information Criterion values indicate improved model fit. Given that the Bayesian Information Criterion tends to favor models with fewer latent classes (37), the Vuong-Lo-Mendell-Rubin likelihood ratio test statistic (38) was also considered. The Vuong-Lo-Mendell-Rubin likelihood ratio test statistic can be used in mixture modeling to compare the fit of the specified class solution (*k*-class model) with that of a model with fewer classes (*k* − 1 class model). A nonsignificant χ^2^ value suggests that a model with 1 fewer class is preferred. Entropy statistics, which measure the separation of the classes based on the posterior class membership probabilities, were also examined; entropy values approaching 1 indicate clear separation between classes (39).

After identifying latent subgroups and assigning subjects to classes based on probability of membership, we used generalized linear models to examine the ways in which experiencing various forms of perceived interpersonal discrimination, experiencing discrimination over time, and experiencing discrimination across multiple domains could place women at risk for depression. Generalized linear models were fitted using the “modified Poisson” approach suggested by Zou (40), which provides relative risks and confidence intervals using robust error variances. Generalized linear models were fitted for all women combined and for each racial/ethnic group separately, to assess how classes of perceived interpersonal discrimination and depressive symptomatology were associated differently across racial/ethnic groups. All models adjusted for marital status, age, nativity, education, occupation, and mental health scores at baseline. Models that assessed the association between perceived interpersonal discrimination and depressive symptomatology for all women combined also adjusted for race/ethnicity. Statistical analyses were conducted using MPlus, version 7 (41), and Stata, version 13 (42) (StataCorp LP, College Station, Texas).

## RESULTS

Four distinct classes of perceived interpersonal discrimination were identified in the latent class analyses (see Table 1). Class characteristics are shown in Web Table 1 (available at https://academic.oup.com/aje). The largest proportion of the sample (34%; class 3: accumulation of several domains over time; attribution due to sex and other reasons) experienced the accumulation of several domains of perceived interpersonal discrimination over time (namely being treated with less courtesy or respect; receiving poorer service; people acting as if the respondent was not smart or as if they were better than the respondent; and being ignored) and attributed their experiences of perceived interpersonal discrimination mainly to sex and other attributes. The second largest class (28%; class 4: accumulation of some domains over time; attribution due to other reasons; reduction over time) experienced accumulation of perceived interpersonal discrimination across some domains (being treated with less courtesy or respect and people acting as if they were better than the respondent), although experiences diminished over time. Attributions in class 4 were to reasons other than race/ethnicity or sex. Class 1 (21%; accumulation of perceived interpersonal discrimination over time, domains, and attributes) captured participants who had experienced the highest accumulation of perceived interpersonal discrimination over time, domains, and attributes. Finally, class 2 (17%; no experiences of perceived interpersonal discrimination) included participants who reported having no experiences or very minimal experiences of perceived interpersonal discrimination across any of the 6 time points.

**Table 1.** Indices of the Fit of Classes of Perceived Interpersonal Discrimination Identified in Latent Class Analysis, Study of Women’s Health Across the Nation, 1996–2008

No. of Classes	Sample-Size-Adjusted BIC	Entropy	Log-Likelihood	Vuong-Lo-Mendell-Rubin Likelihood Ratio Test (Δ)	*P* for Δ^a^
2	184,538.216	0.962	−91,897.863		
3	175,636.544	0.941	−87,260.206	9,260.412	0.0000
4	172,540.157	0.920	−85,525.192	3,464.453	0.0000
5	171,291.239	0.903	−84,713.913	1,619.952	0.4688

Abbreviation: BIC, Bayesian Information Criterion.

^a^*P* value for the likelihood ratio test.

Table 2 shows the distribution of sociodemographic characteristics and outcomes of women in the SWAN study across the 4 distinct classes of perceived interpersonal discrimination. Class 1 had the highest proportions of African-American (41%) and Chinese-American (18%) women compared with any other class, whereas class 2 had the highest proportions of non-Hispanic white (64%) and Japanese-American (19%) women. The distributions of educational qualifications were similar across classes. Class 1 had the highest proportions of single and divorced women (17% and 23%, respectively), and married women were overrepresented in class 2 (76%). Class 1 had the highest mental health score at baseline and at wave 10, whereas class 2 had the lowest mental health score at both time points.

**Table 2.** Sociodemographic Characteristics (%) of the Study Sample According to Latent Class of Perceived Interpersonal Discrimination, Study of Women’s Health Across the Nation, 1996–2008

Characteristic	Class			
	Class 1^a^ (*n* = 366)	Class 2^b^ (*n* = 203)	Class 3^c^ (*n* = 561)	Class 4^d^ (*n* = 483)
Age, years^e^	45.9 (2.6)	46.2 (2.8)	45.8 (2.6)	45.8 (2.7)
Race/ethnicity				
Black or African-American	40.7	10.3	25.7	20.1
Chinese-American	17.8	5.9	12.5	7.5
Japanese-American	9.8	19.2	9.8	10.8
Non-Hispanic white	31.7	64.5	52.1	61.7
Education				
Less than high school	2.7	1.5	2.9	2.5
High school graduate	12.6	13.8	12.7	14.9
Some college/technical school	33.1	30.5	31.7	32.7
College graduate	22.7	23.7	24.8	20.5
Postgraduate education	29.0	30.5	28.0	29.4
Marital status				
Single or never married	16.9	9.4	13.6	14.3
Married	60.1	75.9	67.7	68.7
Separated, widowed, or divorced	23.0	14.8	18.7	17.0
Nativity				
Born abroad	15.6	24.6	15.9	15.7
Born in the United States	84.4	75.4	84.1	84.3
Occupation				
Professional	56.8	58.6	60.4	58.8
Nonmanual worker	6.0	7.4	5.4	6.6
Skilled manual worker	23.0	21.2	17.8	19.9
Semiskilled or unskilled manual worker	14.2	12.8	16.4	14.7
CES-D score at baseline^e^	0.61 (0.49)	0.33 (0.47)	0.51 (0.50)	0.46 (0.50)
CES-D score at wave 10^e^	0.57 (0.50)	0.25 (0.43)	0.46 (0.50)	0.36 (0.48)

Abbreviation: CES-D, Center for Epidemiologic Studies Depression Scale.

^a^ Accumulation of perceived discrimination over time, domains, and attributes.

^b^ No experiences of perceived interpersonal discrimination.

^c^ Accumulation of several domains over time; attribution due to sex and other reasons.

^d^ Accumulation of some domains over time; attribution due to other reasons; reduction over time.

^e^ Values are expressed as mean (standard deviation).

Table 3 presents the descriptors of latent class membership according to relevant covariates. Older women were less likely than younger women to be in class 4 versus class 2 (odds ratio = 0.75, 95% confidence interval (CI): 0.60, 0.95). Odds of membership in any of the classes that captured different types of cumulative perceived interpersonal discrimination (class 1, 3, or 4) were much higher for African-American and Chinese-American women than for non-Hispanic white women. In the case of membership in class 1 compared with class 2, odds of membership for African-American and Chinese-American women were about 6 times those of non-Hispanic white women (see Table 3). Women with a high level of education and women born in the United States were more likely than their counterparts to be in class 1, 3, or 4 than in class 2. Women who were married were less likely than single women to be in class 1, 3, or 4 than in class 2, and so were women with semiskilled and unskilled occupations, compared with professional women.

**Table 3.** Odds Ratios for Membership in a Latent Class Involving Perceived Interpersonal Discrimination as Compared With Class 2 (No Experiences of Perceived Discrimination), According to Sociodemographic Characteristics Relevant to Attributions of Perceived Discrimination, Study of Women’s Health Across the Nation, 1996–2008

Characteristic	Latent Class Comparison					
	Class 1^a^ vs. Class 2^b^		Class 3^c^ vs. Class 2		Class 4^d^ vs. Class 2	
	OR	95% CI	OR	95% CI	OR	95% CI
Age, years						
40–45	1.00	Referent	1.00	Referent	1.00	Referent
≥46	0.82	0.64, 1.05	0.86	0.69, 1.07	0.75	0.60, 0.95
Race/ethnicity						
Non-Hispanic white	1.00	Referent	1.00	Referent	1.00	Referent
Black or African-American	6.29	4.37, 9.05	2.38	1.69, 3.36	1.48	1.03, 2.12
Chinese-American	5.87	3.31, 10.41	2.65	1.53, 4.61	1.39	0.77, 2.49
Japanese-American	0.87	0.57, 1.32	0.49	0.34, 0.71	0.49	0.34, 0.71
Education						
Less than high school	1.00	Referent	1.00	Referent	1.00	Referent
High school graduate	4.74	2.44, 9.21	3.25	2.02, 5.24	2.55	1.60, 4.07
Some college/technical school	7.61	4.05, 14.28	3.96	2.53, 6.20	3.38	2.19, 5.22
College graduate	8.58	4.47, 16.46	4.98	3.10, 7.99	3.11	1.95, 4.97
Postgraduate education	8.37	4.38, 15.99	5.36	3.36, 8.55	3.60	2.27, 5.68
Marital status						
Singe or never married	1.00	Referent	1.00	Referent	1.00	Referent
Married	0.36	0.24, 0.53	0.60	0.41, 0.88	0.59	0.40, 0.87
Separated, widowed, or divorced	0.71	0.44, 1.13	0.86	0.55, 1.36	0.72	0.45, 1.15
Nativity						
Born abroad	1.00	Referent	1.00	Referent	1.00	Referent
Born in the United States	4.72	3.48, 6.39	4.42	3.41, 5.72	3.09	2.40, 3.99
Occupation						
Professional	1.00	Referent	1.00	Referent	1.00	Referent
Nonmanual worker	0.64	0.37, 1.12	0.58	0.35, 0.95	0.70	0.42, 1.16
Skilled manual worker	1.07	0.75, 1.53	0.91	0.65, 1.27	0.95	0.68, 1.33
Semiskilled or unskilled worker	0.55	0.38, 0.80	0.61	0.44, 0.84	0.58	0.41, 0.81

Abbreviations: CI, confidence interval; OR, odds ratio.

^a^ Accumulation of perceived discrimination over time, domains, and attributes.

^b^ No experiences of perceived interpersonal discrimination.

^c^ Accumulation of several domains over time; attribution due to sex and other reasons.

^d^ Accumulation of some domains over time; attribution due to other reasons; reduction over time.

Compared with women who experienced the highest accumulation of perceived interpersonal discrimination (class 1), women in all other classes tended to be less likely to report depressive symptomatology (see Table 4). This was true across all racial/ethnic groups. For example, compared with African-American women in class 1, African-American women in classes 2 and 4 had 0.46 and 0.65 times the risk, respectively, of reporting depression (Table 4). Chinese-American women in classes 2, 3, and 4, who experienced less perceived interpersonal discrimination over time, domains, and attributes, were all less likely to report depression than Chinese-American women in class 1. Japanese-American women in class 3 (accumulation of several domains over time; attribution due to sex and other reasons) had 0.65 times the risk of depression, compared with women in class 1 (incidence rate ratio = 0.65, 95% CI: 0.42, 1.00). Non-Hispanic white women in class 2 or class 4 were 0.52 and 0.69 times as likely, respectively, to report depression compared with women in class 1 (see Table 4). When pooled together, women of all racial/ethnic groups in classes 2, 3, and 4 had a lower risk of depression than women in class 1. This was particularly strong for women who reported the lowest levels of perceived interpersonal discrimination (class 2), who had 0.46 times the risk of depression compared with women with the highest levels of cumulative experiences of perceived interpersonal discrimination, in class 1 (incidence rate ratio = 0.46, 95% CI: 0.35, 0.59).

**Table 4.** Association Between Latent Class of Perceived Interpersonal Discrimination and Depression^a^, Study of Women’s Health Across the Nation, 1996–2008

Class	Race/Ethnicity									
	Black or African-American (*n* = 411)		Chinese-American (*n* = 183)		Japanese-American (*n* = 182)		Non-Hispanic White (*n* = 837)		All Women (*n* = 1,613)	
	IRR^b^	95% CI	IRR^b^	95% CI	IRR^b^	95% CI	IRR^b^	95% CI	IRR^c^	95% CI
Class 1^d^	1.00	Referent	1.00	Referent	1.00	Referent	1.00	Referent	1.00	Referent
Class 2^e^	0.46	0.21, 1.00	1.72*e*^−07^	8.43*e*^−08^, 3.49*e*^*−*07^	0.55	0.28, 1.09	0.52	0.37, 0.72	0.46	0.35, 0.59
Class 3^f^	0.81	0.64, 1.04	0.50	0.30, 0.83	0.65	0.42, 1.00	0.96	0.80, 1.16	0.80	0.71, 0.91
Class 4^g^	0.65	0.47, 0.90	0.56	0.32, 0.98	0.76	0.50, 1.16	0.69	0.56, 0.85	0.64	0.55, 0.74

Abbreviations: CES-D, Center for Epidemiologic Studies Depression Scale; CI, confidence interval; IRR, incidence rate ratio.

^a^ Depression was defined as a CES-D score ≥16.

^b^ Adjusted for marital status, age, nativity, occupation, education, and CES-D score at baseline.

^c^ Adjusted for marital status, age, nativity, occupation, education, CES-D score at baseline, and race/ethnicity.

^d^ Accumulation of perceived discrimination over time, domains, and attributes.

^e^ No experiences of perceived interpersonal discrimination.

^f^ Accumulation of several domains over time; attribution due to sex and other reasons.

^g^ Accumulation of some domains over time; attribution due to other reasons; reduction over time.

## DISCUSSION

### Findings

This study aimed to examine the association between cumulative exposure to perceived interpersonal discrimination over time, attributes, and domains and depression among older women. Drawing from intersectionality theory, we explored how multiple marginalizations and oppressions that women embody over time put them at increased risk of depression at older ages.

We found 4 distinct classes of perceived interpersonal discrimination, ranging from increased cumulative experiences of perceived interpersonal discrimination over time, across all domains, and across attributes to none or very minimal reports of perceived discrimination. Only a minority of the sample (17%) reported having no experiences or minimal experiences of perceived discrimination, and the large majority of women experienced perceived discrimination that was attributed to multiple social identities. Through the 6 waves of data that collected information on experienced perceived discrimination, only 2 women (out of 1,613 participants who had complete data for all 6 waves) reported experiencing only racial discrimination, and only 1 reported experiencing only sex discrimination. This highlights the need to consider multiple social positions and oppressed identities when reporting the prevalence of perceived interpersonal discrimination and understanding the harmful effect of perceived interpersonal discrimination on health. Assessing 1 sole attribution of discrimination does not accurately represent the lived experiences of marginalized populations, who very often experience multiple forms of discrimination. Likewise, we found that very few participants (14 women) had experienced perceived interpersonal discrimination in 1 domain across waves or at 1 time point only (29 women). Studies that measure a single attribute, 1 domain, or 1 point in time underestimate the frequency and complexity of discrimination and its association with health.

Women in class 1 (highest accumulation of perceived interpersonal discrimination) experienced the highest risk of depression compared with women in all other classes, especially compared with women in class 2 (lowest prevalence of perceived interpersonal discrimination). In previous studies, investigators have reported similar findings whereby exposure to multiple forms of discrimination is associated with significantly more depressive symptoms (15, 21), although those studies did not consider multiple domains or time points. Cumulative disadvantage theory and related models such as cumulative advantage and cumulative inequality theory (43) suggest that populations experience health outcomes and trajectories as a result of advantages or disadvantages experienced across the life course. In these analyses of the accumulation of perceived interpersonal discrimination through a period in the later stages of women’s life course, we found clear evidence of the corrosive incremental association that cumulative exposure to disadvantage, in the shape of experienced perceived interpersonal discrimination across attributes, domains, and time, has with mental health.

We found that women with higher education, single women, and US-born women had greater odds of membership in class 1 compared with their noneducated, married, and foreign-born counterparts. These sociodemographic patterns in reports of discrimination have been previously reported in the literature (44–46). Patterns in the association between perceived interpersonal discrimination and mental health were similar across racial/ethnic groups, with some minor differences. This indicates that experiencing perceived discrimination is harmful for women regardless of racial/ethnic background, although the type and severity of discrimination (and therefore, the accumulated harm over the life course) differs across racial/ethnic groups. In our sample, African-American and Chinese-American women had the highest likelihood of membership in class 1 compared with non-Hispanic white women. These differences in class membership reflect that the fact that although marginalized groups, such as women, face more discrimination than privileged groups, individuals who belong to multiple stigmatized groups, such as racial/ethnic minority women and/or women with several marginalized identities, face the greatest burden of these experiences (15, 21).

Japanese-American women’s mental health did not differ between participants in class 2 (lowest prevalence of perceived interpersonal discrimination) and participants in class 1 (highest accumulation of perceived interpersonal discrimination), although we found significant differences across these classes for the other racial/ethnic groups. Class 2 does not fully capture women who have never experienced any discrimination—most participants in class 2 reported very few experiences of perceived discrimination either due to 1 attribute, in 1 domain, or at 1 time point. Some studies show a J-shaped relationship between racism and health such that people who report experiencing no racism still have poor health (47), perhaps because lack of reporting does not necessarily mean that people have not experienced any discrimination but may mean that they deny these experiences as a self-defense mechanism. It is therefore possible that the lack of differences in risk of mental health found between classes 1 and 2 is due to this J-shaped curve association between perceived discrimination and health.

### Limitations

Although this study was able to take advantage of the longitudinal and multidimensional nature of SWAN data, it was limited in some respects. First, SWAN does not ask respondents about exposure to perceived discrimination over the course of their lives, so we were unable to examine any of the processes or experiences of perceived discrimination prior to their baseline interview. We also did not have any data on vicarious exposure to discrimination or data on internalized systems of oppression, such as internalized racism and sexism, which have been shown to be detrimentally associated with poor health (29, 48). Furthermore, even though we are able to examine experiences across various domains of perceived discrimination, the domains explored do not represent the full range of places and circumstances where discrimination can be experienced. Given these measurement limitations, results presented here may underestimate the prevalence of accumulated discrimination and its association with mental health.

This study focused on women only, and although these associations may be similar in men, we are not able to assert that here. In general, women are more likely than men to experience multiple forms of discrimination, since they are part of an oppressed social group and may embody other social identities that subject them to discrimination due to other attributes. For example, the accumulation of racism and sexism that racial/ethnic minority women experience in their lifetimes contributes to high levels of stress and psychiatric symptoms, but the majority of research studies focus on either race-related or sex-related stress and do not capture the ways in which racial/ethnic minority women experience both race- and sex-related stress simultaneously (49); we aimed to achieve that in this study. Applying latent class analysis to SWAN data allowed us to show how additive associations with different attributes of perceived interpersonal discrimination and multiple marginalization, over time and domains, worsen the detrimental association between discrimination and health. However, we were not able to fully examine how the interrelation and interaction between 2 attributes combined into 1 specific form of discrimination, such as gendered racism, is associated with women’s mental health. Future studies employing other statistical techniques may be able to consider in greater detail the embodied positions of multiply discriminated-against individuals and their associations with health.

### Conclusions

This study documents, for the first time, the harm that cumulative experiences of perceived interpersonal discrimination over attributes, domains, and time have on the mental health of ethnic minority women. We found that women of all racial/ethnic minority groups who experience the highest levels of cumulative perceived discrimination are at greater risk of depression than women who experience minimal levels of perceived discrimination that do not accumulate over time, attributes, or domains. Although we found that experiencing perceived interpersonal discrimination is harmful for women regardless of racial/ethnic background, our results show that the type and severity of perceived discrimination differs across racial/ethnic groups, and it is more harmful for racial/ethnic minority women.

The findings of this study highlight the need to fully capture the experiences of discrimination of marginalized populations, in order to avoid underestimating the magnitude and complexity of discrimination and its association with health.

## Supplementary Material

Web Material

## Abbreviations

CI: confidence interval
SWAN: Study of Women’s Health Across the Nation
