# Supplementary material for: Perceived Interpersonal Discrimination and Older Women’s Mental Health: Accumulation Across Domains, Attributions, and Time
Source: Am J Epidemiol. 2017 Oct 4;187(5):924–32. doi: 10.1093/aje/kwx326 (PMC5928463; doi:10.1093/aje/kwx326)
Supplement: Web Material [file webtable1.docx]

**Web Table 1. Latent Classes of Perceived Interpersonal Discrimination, Study of Women’s Health Across the Nation (1996-2008)**

| **Observed Variables** | **Class 1**^a^  (n=597) | **Class 2**^b^  (n=496) | **Class 3**^c^  (n=979) | **Class 4**^d^  (n=814) |
| --- | --- | --- | --- | --- |
| Treated with less courtesy than others - Baseline | 0.993 | 0.324 | 0.936 | 0.732 |
| Treated with less respect than others - Baseline | 0.988 | 0.262 | 0.935 | 0.709 |
| Receives poorer service than others - Baseline | 0.972 | 0.153 | 0.866 | 0.567 |
| People act as if they think you are not smart - Baseline | 0.955 | 0.134 | 0.824 | 0.495 |
| People act as if they are afraid of you - Baseline | 0.825 | 0.140 | 0.550 | 0.356 |
| People act as if they think you are dishonest - Baseline | 0.752 | 0.030 | 0.361 | 0.158 |
| People act as if they're better than you are - Baseline | 0.989 | 0.279 | 0.904 | 0.702 |
| You or your family members are called names/insulted - Baseline | 0.793 | 0.038 | 0.352 | 0.184 |
| You are threatened or harassed - Baseline | 0.680 | 0.029 | 0.301 | 0.158 |
| People ignore you or act as if you are not there - Baseline | 0.942 | 0.109 | 0.732 | 0.429 |
| Treated with less courtesy than others - Wave 1 | 0.982 | 0.221 | 0.944 | 0.691 |
| Treated with less respect than others - Wave 1 | 0.981 | 0.200 | 0.943 | 0.679 |
| Receives poorer service than others - Wave 1 | 0.962 | 0.115 | 0.875 | 0.54 |
| People act as if they think you are not smart - Wave 1 | 0.965 | 0.106 | 0.832 | 0.524 |
| People act as if they are afraid of you - Wave 1 | 0.882 | 0.130 | 0.603 | 0.399 |
| People act as if they think you are dishonest - Wave 1 | 0.760 | 0.014 | 0.418 | 0.184 |
| People act as if they're better than you are - Wave 1 | 0.982 | 0.190 | 0.931 | 0.665 |
| You or your family members are called names/insulted - Wave 1 | 0.754 | 0.036 | 0.361 | 0.159 |
| You are threatened or harassed - Wave 1 | 0.640 | 0.012 | 0.271 | 0.118 |
| People ignore you or act as if you are not there - Wave 1 | 0.922 | 0.060 | 0.761 | 0.378 |
| Treated with less courtesy than others - Wave 2 | 0.991 | 0.187 | 0.923 | 0.659 |
| Treated with less respect than others - Wave 2 | 0.992 | 0.146 | 0.917 | 0.619 |
| Receives poorer service than others - Wave 2 | 0.973 | 0.093 | 0.834 | 0.459 |
| People act as if they think you are not smart - Wave 2 | 0.955 | 0.093 | 0.789 | 0.480 |
| People act as if they are afraid of you - Wave 2 | 0.840 | 0.113 | 0.561 | 0.332 |
| People act as if they think you are dishonest - Wave 2 | 0.791 | 0.013 | 0.362 | 0.132 |
| People act as if they're better than you are - Wave 2 | 0.983 | 0.203 | 0.883 | 0.592 |
| You or your family members are called names/insulted - Wave 2 | 0.755 | 0.021 | 0.266 | 0.117 |
| You are threatened or harassed - Wave 2 | 0.650 | 0.025 | 0.208 | 0.095 |
| People ignore you or act as if you are not there - Wave 2 | 0.945 | 0.057 | 0.678 | 0.334 |
| Treated with less courtesy than others - Wave 3 | 0.986 | 0.171 | 0.925 | 0.574 |
| Treated with less respect than others - Wave 3 | 0.991 | 0.152 | 0.920 | 0.556 |
| Receives poorer service than others - Wave 3 | 0.968 | 0.086 | 0.818 | 0.368 |
| People act as if they think you are not smart - Wave 3 | 0.937 | 0.112 | 0.795 | 0.410 |
| People act as if they are afraid of you - Wave 3 | 0.844 | 0.088 | 0.558 | 0.263 |
| People act as if they think you are dishonest - Wave 3 | 0.795 | 0.015 | 0.360 | 0.106 |
| People act as if they're better than you are - Wave 3 | 0.977 | 0.175 | 0.894 | 0.519 |
| You or your family members are called names/insulted - Wave 3 | 0.741 | 0.020 | 0.287 | 0.090 |
| You are threatened or harassed - Wave 3 | 0.627 | 0.010 | 0.231 | 0.074 |
| People ignore you or act as if you are not there - Wave 3 | 0.925 | 0.065 | 0.679 | 0.281 |
| Treated with less courtesy than others - Wave 7 | 0.983 | 0.160 | 0.892 | 0.559 |
| Treated with less respect than others - Wave 7 | 0.981 | 0.107 | 0.897 | 0.531 |
| Receives poorer service than others - Wave 7 | 0.941 | 0.053 | 0.770 | 0.381 |
| People act as if they think you are not smart - Wave 7 | 0.943 | 0.105 | 0.770 | 0.433 |
| People act as if they are afraid of you - Wave 7 | 0.818 | 0.061 | 0.487 | 0.245 |
| People act as if they think you are dishonest - Wave 7 | 0.726 | 0.012 | 0.300 | 0.098 |
| People act as if they're better than you are - Wave 7 | 0.965 | 0.159 | 0.862 | 0.521 |
| You or your family members are called names/insulted - Wave 7 | 0.627 | 0.021 | 0.239 | 0.096 |
| You are threatened or harassed - Wave 7 | 0.585 | 0.023 | 0.206 | 0.086 |
| People ignore you or act as if you are not there - Wave 7 | 0.889 | 0.067 | 0.618 | 0.320 |
| Treated with less courtesy than others - Wave 10 | 0.963 | 0.069 | 0.805 | 0.494 |
| Treated with less respect than others - Wave 10 | 0.971 | 0.069 | 0.798 | 0.463 |
| Receives poorer service than others - Wave 10 | 0.906 | 0.039 | 0.681 | 0.294 |
| People act as if they think you are not smart - Wave 10 | 0.922 | 0.055 | 0.690 | 0.329 |
| People act as if they are afraid of you - Wave 10 | 0.745 | 0.066 | 0.369 | 0.218 |
| People act as if they think you are dishonest - Wave 10 | 0.678 | 0.007 | 0.239 | 0.082 |
| People act as if they're better than you are - Wave 10 | 0.932 | 0.098 | 0.778 | 0.444 |
| You or your family members are called names/insulted - Wave 10 | 0.607 | 0.015 | 0.177 | 0.083 |
| You are threatened or harassed - Wave 10 | 0.534 | 0.005 | 0.149 | 0.070 |
| People ignore you or act as if you are not there - Wave 10 | 0.872 | 0.055 | 0.584 | 0.247 |
| Attribution to race - Baseline | 0.451 | 0.292 | 0.234 | 0.226 |
| Attribution to gender - Baseline | 0.091 | 0.047 | 0.181 | 0.161 |
| Attribution to race - Wave 1 | 0.540 | 0.007 | 0.228 | 0.082 |
| Attribution to gender - Wave 1 | 0.511 | 0.005 | 0.291 | 0.102 |
| Attribution to race - Wave 2 | 0.488 | 0.024 | 0.218 | 0.072 |
| Attribution to gender - Wave 2 | 0.495 | 0.007 | 0.282 | 0.080 |
| Attribution to race - Wave 3 | 0.493 | 0.031 | 0.189 | 0.061 |
| Attribution to gender - Wave 3 | 0.496 | 0.006 | 0.261 | 0.074 |
| Attribution to race - Wave 7 | 0.593 | 0.116 | 0.344 | 0.162 |
| Attribution to gender - Wave 7 | 0.641 | 0.344 | 0.469 | 0.244 |
| Attribution to race - Wave 10 | 0.606 | 0.086 | 0.306 | 0.218 |
| Attribution to gender - Wave 10 | 0.582 | 0 | 0.468 | 0.230 |
| Other Attribution - Wave 1 | 0.585 | 0.041 | 0.417 | 0.212 |
| Other Attribution - Wave 2 | 0.548 | 0.061 | 0.402 | 0.177 |
| Other Attribution - Wave 3 | 0.579 | 0.045 | 0.383 | 0.145 |
| Other Attribution - Wave 7 | 0.768 | 0.597 | 0.765 | 0.742 |
| Other Attribution - Wave 10 | 0.811 | 0.806 | 0.730 | 0.745 |

^a^ Class 1: Accumulation of perceived discrimination over time, domains and attributions

^b^ Class 2: No experiences of perceived interpersonal discrimination

^c^ Class 3: Accumulation of several domains over time. Attribution due to gender and other reasons

^d^ Class 4: Accumulation of some domains over time. Attribution due to other reasons. Reduction over time
